# Advances in Reducing Salt Content in Processed Meats with Basic Amino Acids

**DOI:** 10.3390/foods14060940

**Published:** 2025-03-10

**Authors:** Rui Fang, Zongshuai Zhu

**Affiliations:** 1 Center for Molecular Metabolism, Nanjing University of Science & Technology, Nanjing 210094, China; fangrui@njust.edu.cn; 2 Key Laboratory of Metabolic Engineering and Biosynthesis Technology, Ministry of Industry and Information Technology, Nanjing 210094, China; 3 School of Food Science and Technology, Henan Institute of Science and Technology, No. 655 Hua Lan Street, Xinxiang 453003, China

**Keywords:** basic amino acids, sodium reduction, meat products, sensory attributes

## Abstract

Basic amino acids have emerged as a pivotal area of research in efforts to decrease the sodium content in meat products, primarily due to their ability to enhance flavor, improve taste, and effectively replace sodium salts. This review synthesizes current strategies for sodium reduction in meat products and offers an overview of previous studies examining the role of basic amino acids in such applications, including their impact on sensory attributes and structural alterations. Furthermore, the implications of these strategies on product quality are examined, addressing aspects such as protein hydrolysis, oxidation, color, and textural changes, as well as potential underlying mechanisms. Additionally, future challenges and trends in the utilization of basic amino acids in processed meats are explored. Overall, basic amino acids exhibit significant potential as sodium salt substitutes, particularly at low NaCl concentrations. Their combinations with chloride salts, yeast extracts, and other salts have been explored as alternative sodium reduction strategies. However, challenges remain in their application to meat products, including high production costs, consumer acceptance, and stability during large-scale production. Future research should focus on optimizing the use of basic amino acids, enhancing their economic feasibility, and addressing technical hurdles.

## 1. Introduction

**Figure 1:**
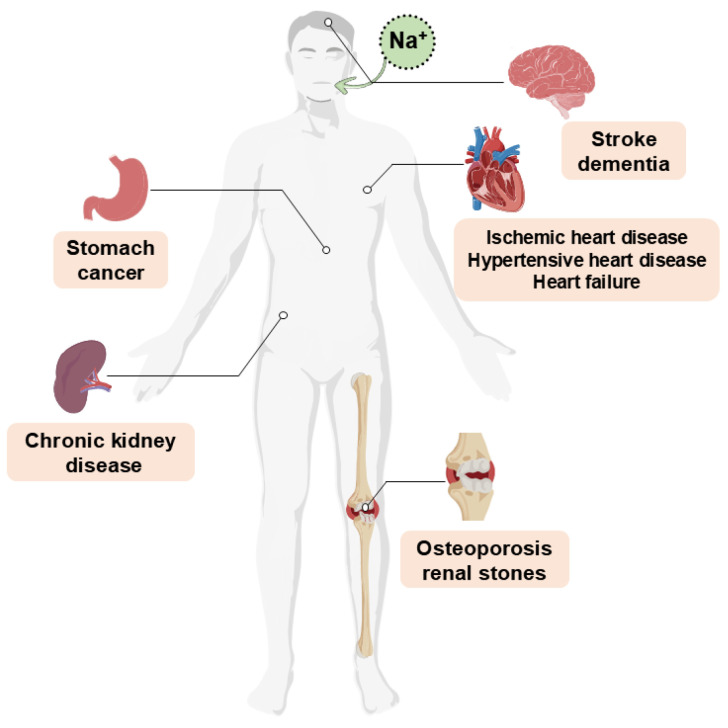
Health risks associated with excessive salt intake.

Salt is a widely used flavoring agent in cooking and the food industry, serving multiple functions, including enhancing food flavor, stimulating appetite, inhibiting microbial growth, extending shelf life, maintaining fluid balance, and regulating physiological functions [1]. The per capita daily salt intake in most countries and regions worldwide ranges from approximately 9 to 12 g, significantly exceeding the World Health Organization (WHO) recommendation of 5 g per day [2]. However, the adverse effects of excessive salt intake are often underestimated. In 2017, high salt consumption was estimated to be responsible for approximately 3 million deaths and 70 million disability-adjusted life years worldwide [3]. A substantial body of research indicates that high-salt diets are strongly linked to non-communicable diseases, contributing not only to the development of hypertension and cardiovascular diseases but also potentially compromising the immune system and inducing autoimmune disorders (Figure 1) [4]. However, the Yanomami people in the Amazon region are virtually free from hypertension due to their low-salt diet, suggesting that reducing salt intake is effective in lowering the risk of hypertension [5]. Furthermore, the prevalence of stroke decreased by 10.1% in Finland and 23.1% in Poland following the implementation of low-salt diets [6]. These success stories have inspired countries to adopt salt reduction strategies aimed at alleviating the health burden associated with excessive salt consumption. In 2012, the WHO included salt reduction as one of the seven pillars of the Global Action Plan for the Prevention of Non-communicable Diseases, with member states committing to a 30% reduction in salt intake by 2025, which is projected to save an estimated 1.65 million lives annually [7]. Implementation strategies in many countries and regions include reformulating food recipes, establishing targets for maximum sodium content in products, implementing labeling regulations or taxes on items with high sodium levels, and conducting active campaigns to raise awareness and educate consumers [1]. Japan estimates that salt reduction policies could prevent 1–3% of cardiovascular events and reduce the associated healthcare costs by up to 2% over a 10-year period beginning in 2019, reflecting modest health and economic benefits [8]. Wang et al. [9] projected that achieving salt reduction targets in China could prevent 197,000 cardiovascular events annually and result in a 2.5% decrease in cardiovascular mortality. Furthermore, this initiative could save approximately 1.4 billion international dollars. It is estimated that the salt reduction initiative in the UK could save approximately 9000 lives each year and reduce healthcare costs by around £1.5 billion annually [10]. A study conducted in the United States revealed that even a modest 10% reduction in salt intake could prevent hundreds of thousands of strokes and heart attacks over the lifetime of adults aged 40 to 85 years, potentially saving more than 32 billion USD in healthcare costs in the U.S. alone [11].

Meat consumption continues to rise, driven by globalization and advancements in cold chain logistics technology. Consequently, meat products have become a significant source of sodium intake, accounting for approximately 20% of daily sodium consumption [12]. While raw meat naturally contains a small amount of sodium, the addition of sodium chloride and sodium salt additives during processing substantially increases its sodium content. Traditional curing techniques utilize high salt concentrations to inhibit water activity, thereby extending shelf life [13,14]. Classic cured and fermented meat products, such as Jinhua ham, Parma ham, and salami, can contain over 10% sodium, underscoring the need to reduce sodium levels in meat products. However, directly reducing sodium not only diminishes the perceived salinity but may also adversely affect tenderness, flavor, gel strength, and the water-holding capacity (WHC), which can promote the growth of spoilage and pathogenic bacteria and limit their applicability in meat processing [15,16,17,18,19]. Therefore, the challenge of sodium without compromising taste or quality has become a critical research issue, highlighting the urgent need for innovative salt substitutes to lower sodium content in processed meat products.

Amino acids serve as common flavor enhancers, and the incorporation of basic amino acids, such as L-lysine (L-Lys), L-histidine (L-His), and L-arginine (L-Arg), as substitutes for NaCl in processed meats can significantly enhance the perception of saltiness. This substitution compensates for potential flavor loss resulting from a reduced salt content [20]. At a pH of 7, these amino acids carry a positive charge due to the presence of an additional amino group, allowing their side chains to interact with oppositely charged groups to form ionic bonds or salt bridges [21]. In food processing, basic amino acids can effectively substitute for some of the functional roles of salt, particularly in meat products and seasonings. They offer superior texture modification in low-salt food products, enhance the overall sensory quality of meat while reducing sodium content, and improve not only the flavor but also the textural properties, particularly increasing the tenderness and elasticity [22]. By decreasing sodium salt usage, basic amino acids help restore or maintain the product’s taste and texture, preventing it from becoming excessively dry, hard, or loose [23]. Zheng et al. [24] found that the addition of appropriate amounts of lysine or arginine significantly reduced cooking loss, improved water retention, and enhanced textural parameters such as hardness, elasticity, and chewiness. Wachirasiri et al. [25] demonstrated that replacing phosphate with L-Lys or L-Arg in the treatment of white shrimp significantly reduced juice loss during the thawing of frozen shrimp. Additionally, several studies have indicated that complex formulations, such as amino acids, can mitigate the adverse effects of partially substituting sodium salts with potassium salts, particularly regarding the deterioration of organoleptic quality. For instance, Turk [26] utilized a mixture of lysine and succinic acid to mask metallic taste, enhance salinity, and improve overall flavor. Dos Santos et al. [27] found that incorporating complex flavor-enhancing substances, such as sodium guanylate, sodium inosinate, monosodium glutamate, and lysine, could alleviate the negative effects of substituting sodium salts with potassium or magnesium salts, thereby reducing the metallic astringent taste associated with potassium salts. Basic amino acids not only function as flavor enhancers but also play a crucial role in other food properties, including antioxidant activity, water retention, and various other functions [28,29]. This makes them valuable additives in salt-reduced foods, with significant potential for widespread application in meat products and seasonings in the future.

This review aims to discuss the existing strategies for reducing sodium in meat products, providing an in-depth analysis of the necessity of decreasing salt levels through the use of basic amino acids, their effects on meat products, applications, and the challenges encountered. Furthermore, a comprehensive overview of the research progress on salt reduction using basic amino acids during meat processing is presented. The objective of this review is to provide insights into the rational design and widespread application of basic amino acids, aiming to reduce sodium content in meat products without compromising flavor and quality.

## 2. Strategies for Reducing Sodium in Meat Products

**Figure 2:**
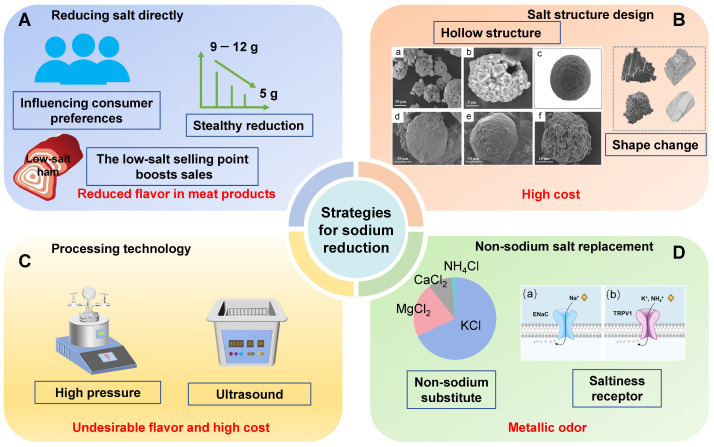
Strategies for reducing sodium in meat products. (**A**) Direct reduction in salt content. (**B**) Design of unique physical structures, including shape alterations and hollow structures (a–f). (**C**) Changes in processing techniques. (**D**) Use of non-sodium alternatives; the main salt taste receptor in human (a: epithelial sodium channel receptor; b: transient receptor potential vanillic acid receptor).

Sodium salts in meat products serve purposes beyond flavor enhancement; they are crucial for preservation and maintaining the structural integrity of the meat. This is especially important for products that require extended storage, such as ham and bacon. Germond et al. [30] argued that these cured meat products not only require storage in caves with low temperatures and constant humidity at high altitudes, but that processors often need to invest in maturation facilities with controlled temperature and humidity. This investment incurs high energy costs due to the prolonged maturation process. Therefore, any strategy aimed at reducing salt content must take into account the product’s shelf life, flavor, and environmental sustainability impact. Currently, salt reduction techniques for meat products can be categorized as follows: direct reduction in sodium chloride (Figure 2A), modification of salt forms (Figure 2B), innovative processing techniques (Figure 2C), and the use of sodium chloride substitutes (Figure 2D).

### 2.1. Gradual Reduction in Salt Content

Gradual and subtle reductions in NaCl concentration in meat products have become one of the most widely employed strategies for salt reduction in the food industry [31]. The effectiveness of this approach stems from the fact that consumers’ preferences for saltiness are not fixed; rather, they are influenced by perceptual, environmental, and behavioral factors [32]. By gradually decreasing sodium content over an extended period, consumers’ palates can adapt to lower sodium formulations, thereby enabling the achievement of higher salt reduction targets. This strategy has yielded significant results in several countries. For instance, the United Kingdom successfully reduced the average sodium intake of its population by 15% between 2000 and 2011 by establishing and monitoring specific salt reduction targets [33].

Coordination within the industry is essential for the gradual reduction in salt in meat products. If only one brand reduces its salt content while others maintain higher sodium levels, consumers may find the salt-reduced products less acceptable. Studies have shown that the consumption of products with higher salt content diminishes consumers’ taste sensitivity, thereby affecting their acceptance of salt-reduced alternatives [34,35]. Therefore, to ensure the success of salt reduction strategies, the meat industry must align its salt reduction targets, facilitating consumers’ gradual adaptation to low-sodium products.

Although this strategy can effectively reduce sodium intake, it may pose time-consuming challenges and certain risks for manufacturers. Initiatives aimed at reducing salt can lead to unpredictable changes in food storage, structure, and flavor characteristics, potentially resulting in economic fluctuations due to shifts in consumer preferences and purchasing behavior [36]. Consequently, the potential for further sodium reduction remains limited, necessitating the exploration of alternative and more effective strategies.

### 2.2. Optimization of Salt Crystal Shape

The perception of salty flavor intensity can be significantly enhanced by modifying the size, morphology, and surface area of salt particles, thereby reducing the overall salt intake without compromising taste. This modification primarily influences the dissolution rate of salt crystals in saliva. Conventional salt crystals are typically cubic and have a particle size of approximately 400 µm; however, research has demonstrated that the size and morphology of salt particles significantly affect both the dissolution rate and the perceived saltiness [37]. For example, Daniela et al. [38] found that smaller NaCl crystals dissolve more rapidly, resulting in a more efficient release of sodium into the saliva and an enhanced salty taste. Furthermore, salt crystals with flaky, dendritic, or hollow pyramidal morphologies also exhibit higher dissolution rates and increased saltiness [37].

A prime example is the SODA-LO^®^ salt microspheres produced by Tate and Lyle (London, UK), which are composed of NaCl and solid organic materials with a particle size of less than 100 microns. These microspheres exhibit excellent flow properties and preserve the perception of salty flavor while reducing the sodium content. Their hollow structure maximizes the specific surface area of the salt, thereby enhancing the intensity of the salty flavor and minimizing any chemical aftertaste in various food applications [14,35,39].

In summary, altering the physical form of salt—such as through the use of structures like flakes, dendrites, or hollow microspheres—represents an effective strategy for reducing salt content. However, despite its significant advantages in reducing salt and enhancing salty flavor perception, there are some drawbacks. For instance, the elevated production costs linked to modified salts, especially the intricate manufacturing processes necessary for hollow and microsphere salts, remain in the early stages of development. There have been relatively few successful instances of spray-dried hollow salts, which necessitate drying tower equipment capable of managing high temperatures and rapid spray rates, thereby restricting their widespread application in commercial food products [40]. Additionally, these modified salts may alter the taste and texture of the product, especially under high-temperature or prolonged storage conditions. Therefore, while modifying the physical form of salt offers new possibilities for salt reduction, its commercial application still faces challenges and limitations.

### 2.3. Advancements in Processing Technology

High Pressure Processing (HPP) and Intense Ultrasound (US) are emerging technologies that help to reduce the sodium content in meat products [8]. HPP applies pressures ranging from 300 to 600 MPa at mild temperatures (<45 °C) to inactivate bacteria while preserving texture and nutritional value. Guillou et al. [41] found that HPP, under low-salt conditions, ensures food safety and extends shelf life. For example, Ferreira et al. [42] utilized HPP to inactivate *Listeria monocytogenes* in emulsified meat products, thereby reducing the risk of microbial contamination. However, the efficacy of HPP in compensating for NaCl reduction is diminished in naturally cured or dry-fermented meat products that do not undergo thermal processing [43]. The global HPP industry is valued at approximately 10 billion USD annually. However, most food products processed using high pressure require cold storage to preserve their organoleptic qualities and may also necessitate aseptic packaging conditions. Both of these factors contribute to increased production costs and entail significant investment and operational expenses [44,45]. US reduces salt concentration while maintaining meat quality by altering muscle fiber structure and enhancing biofilm permeability [46]. For instance, Liu et al. [47] improved the tenderness and low-sodium characteristics of beef through ultrasound-assisted low-sodium salt curing. Although ultrasound treatments effectively reduce sodium content and enhance tenderness, their practical application must consider the potential challenges, such as uniformity issues, undesirable flavors, and high costs. According to the U.S. Food and Drug Administration (FDA), several key factors must be considered to address the gaps in the available safety information regarding ultrasound-treated products. These factors include the amplitude of the applied ultrasound waves, the duration of microorganism exposure to the ultrasound treatment, the specific microorganisms being investigated, the quantity of the food processed, the composition of the food, and the temperature of the treatment applied [12].

### 2.4. Sodium Salt Substitutes

Replacing sodium with other inorganic salts, such as KCl, MgCl_2_, CaCl_2_, and NH₄Cl, is a common method for reducing the salt content, as these cations exhibit antimicrobial effects similar to those of Na⁺ during meat processing [18]. However, the use of a single inorganic salt often results in a fishy or bitter taste [48]. The partial replacement of NaCl with other inorganic salts can enhance the quality of meat products; however, careful attention must be given to the overall concentration of these salts in practical applications. In addition, the high manufacturing costs associated with products containing KCl, along with the potential adverse effects of excessive potassium intake and hyperkalemia on renal function, may raise concerns. For healthy adults, the recommended potassium intake of 3400 mg per day is only slightly higher than the advised sodium limit of 2300 mg per day [49].

## 3. Salt Reduction Potential of Basic Amino Acids

L-Lys, L-Arg, and L-His are essential or semi-essential amino acids characterized by basic side chains. These amino acids play a significant role in food science and biochemistry due to their unique structures. L-Lys features an epsilon-amino group in its side chain, which imparts a positive charge at physiological pH (~7.4). This positive charge enables L-Lys to form ionic bonds with negatively charged regions of proteins, such as carboxyl groups, thereby enhancing protein solubility and stability [50]. Additionally, it contributes to savory flavor perception and improves the texture of meat products. L-Arg contains a guanidine group in its side chain, which contributes to its higher alkalinity and facilitates various intermolecular interactions, including the formation of salt bridges with anions [51]. Additionally, it enhances the WHC and gelation properties of proteins, thereby optimizing food texture. Similarly, L-His features an imidazole group in its side chain, enabling it to be positively charged at neutral pH and to function as a proton donor or acceptor [52]. This characteristic endows L-His with significant buffering, metal ion chelation, and antioxidant properties. These structural attributes not only enhance the savory flavor of meat products, providing both organoleptic and nutritional benefits, but also facilitate new research directions in the development of low-sodium health foods. Compared to traditional techniques, basic amino acids preserve more effectively the texture and flavor of meat products, are less expensive, and offer greater adaptability compared to non-traditional methods. Furthermore, their antioxidant properties and potential health benefits position them as a valuable option in the development of future low-sodium meat products. Table 1 summarizes the effects of partially replacing NaCl with basic amino acids in processed meat products.

## 4. Effect of Basic Amino Acids on the Quality of Meat Products

**Figure 3:**
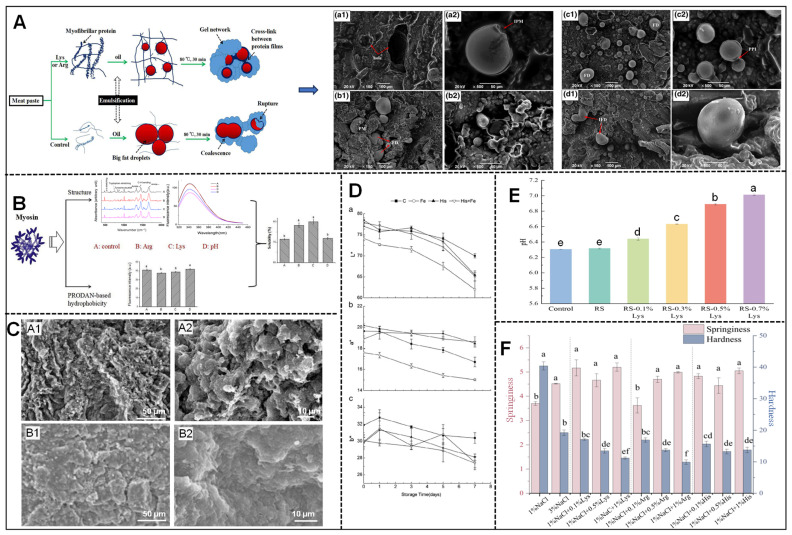
(**A**) The effect of L-Lys and L-Arg on the emulsion stability of meat products [66]; (a1,a2) control, (b1,b2) 0.6% Lys-treated emulsified sausage, (c1,c2) 0.6% Arg-treated emulsified sausage and (d1,d2) 3.0% soybean isolate protein-treated emulsified sausage. (**B**) Spectroscopic techniques and chemical probes used to investigate the mechanisms by which L-Arg and L-Lys influence myosin solubility [63]; different letters (a,b) denote significant differences (*p* < 0.05). (**C**) The impact of L-Arg on the microstructure of chicken actinoglobulin gels [65]; (A1,A2) SEM of Arg 0%/pH 7.23 group and (B1,B2) SEM of Arg 0.1%/pH 7.23 group. (**D**) The influence of L-His on the color changes in meat during storage [68]; (a–c) color changes during storage, respectively. (**E**) pH values of low-sodium frankfurter sausages with varying levels of L-Lys added [54]; different letters (a–e) denote significant differences (*p* < 0.05). (**F**) The effect of basic amino acids on the elasticity of fried beef patties [58]; different letters (a–f) denote significant differences (*p* < 0.05).

Through an in-depth study of the effects on the quality characteristics of meat products, it has been determined that basic amino acids provide several advantages in enhancing the quality of low-sodium meat products. Specifically, lysine and arginine improve the water retention, texture, and emulsification properties (Figure 3A). This enhancement is primarily attributed to arginine’s ability to inhibit protein aggregation [61,62] and the significant increase in myosin solubility facilitated by both lysine and arginine [63] (Figure 3B). Furthermore, these amino acids enhance the water retention and texture of salt-soluble protein gels [64,65] (Figure 3C), underscoring their multifaceted roles in improving the quality of meat products. Zhu et al. [66] investigated the effects of lysine and arginine on the physicochemical properties of chicken intestines and found that both amino acids significantly reduced the total mass percentage of effluent liquid and fat while increasing the mass percentage of protein. Moreover, they improved the emulsification stability of chicken sausage and enhanced its hardness, elasticity, and chewiness. Wen et al. [67] demonstrated that lysine effectively masked the bitter taste of potassium ions, thereby improving the perceived saltiness of sausages. Zhou et al. [68,69] showed that histidine and arginine could inhibit hemoglobin oxidation, thereby stabilizing the color of hemoglobin concentrates (Figure 3D). Wachirasiri et al. [25] found that lysine and arginine improved the color of frozen shrimp during the thawing process. These studies collectively suggest that basic amino acids can improve the various physicochemical properties of meat products, such as pH (Figure 3E), water retention, texture (Figure 3F), color, and flavor. As such, they hold great potential as sodium salt substitutes and may contribute to the further development of low-sodium meat products.

## 5. Molecular Mechanisms of Basic Amino Acids in Flavor Regulation

**Figure 4:**
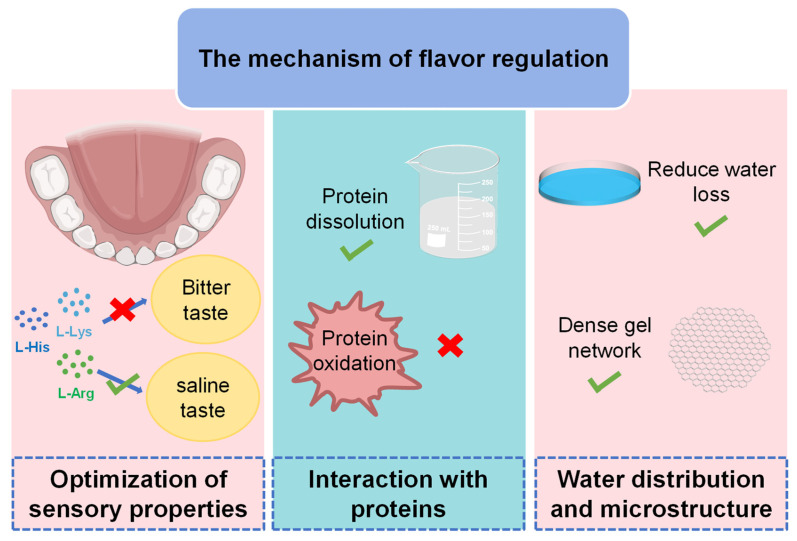
Molecular mechanisms of basic amino acids in flavor regulation (√ denotes promotion, and × denotes inhibition).

Individual amino acids possess distinct flavors and can function independently or synergistically. L-Lys, L-His, and L-Arg exhibit salty, slightly acidic, or bitter notes, yet they can enhance the perception of saltiness in low-salt meat products through taste-gustatory interactions. To further elucidate the specific mechanisms by which basic amino acids influence taste in low-salt meat products, three key perspectives are explored as follows: (1) the modulation of salinity perception, wherein basic amino acids enhance salty flavors and suppress off-flavors, contributing to a more balanced taste profile; (2) the molecular interactions between basic amino acids and other components, such as salts and proteins, within meat products; and (3) the potential for basic amino acids to improve the sensory experience by modulating water distribution and altering the microstructure of products (Figure 4).

### 5.1. Optimization of Sensory Properties

Taste perception relies on the binding of taste-active compounds to specific receptors located in the taste buds. The perception of salty taste is mediated through neurotransduction, which is initiated by the interaction of sodium ions or other ions with salt taste receptors present on the cells of the taste buds [1]. Research has indicated that basic amino acids may exhibit a slightly acidic or bitter flavor when isolated; however, within a certain concentration range, their combination with salt can significantly elevate the threshold for a salty flavor perception, thereby enhancing the saltiness of products under low-salt conditions [70]. Breslin, Harmon, and their colleagues investigated the interactions between substances with savory, acidic, and bitter flavors. They concluded that salty and acidic substances mutually enhance each other’s flavor intensity at low concentrations while inhibiting one another at higher concentrations. Furthermore, the salty taste was found to significantly inhibit the bitter taste [71,72]. Numerous studies have demonstrated that the use of KCl and MgCl_2_ as substitutes for NaCl negatively impacts flavor [73,74,75]. However, this issue can be alleviated by partially replacing NaCl with L-His, L-Lys, and L-Arg, likely due to their unique taste properties, which provide multilevel taste modulation in low-salt meat products. Specifically, the basic amino acids exhibit synergistic effects in enhancing the salty taste. Felicio et al. [76] found that L-Arg could also mask off-flavors, such as bitterness or fishiness, through gustatory-taste interactions, thereby improving the overall flavor balance of the product. The patented salt substitute Pansalt^®^, which contains L-Lys hydrochloride, enhances the salty taste of alternative salts, masks the off-flavors associated with potassium and magnesium salts, and promotes the metabolism of sodium ions in the body [77]. Additionally, basic amino acids may modulate the perception of salty taste through their chemical properties and interactions with taste receptors. For instance, L-Arg, which contains a guanidino group in its side chain, can effectively interact with transmembrane channel-like 4 (TMC4) receptor proteins located in the posterior lingual taste buds, which are involved in the perception of salty flavors, through site-specific hydrogen bonding [78,79]. This interaction not only enhances the perception of salty flavors but may also reduce the need for sodium ions, thereby supporting the health benefits of a low-sodium diet. However, research on the mechanisms by which basic amino acids contribute to sensory enhancement is limited compared to that of metal salts. Further studies are necessary to deepen our understanding of the sensory pathways involved in amino acids and their interactions with other salts.

### 5.2. Protein Interactions

Basic amino acids can modulate the functional expression of salts and proteins in meat products through various intermolecular interactions. Firstly, the side chains of these amino acids are positively charged at physiological pH, allowing them to form ionic bonds with the anionic components of NaCl present in meat products. This interaction enhances the ionic strength of the solution, promoting protein solubilization and expansion [80]. For instance, Takai demonstrated that both Lys and Arg exhibited synergistic effects in solubilizing porcine myosin in a physiological salt solution at pH 7.5 [81]. Guo hypothesized that Lys induces the unfolding of myosin, exposing buried hydrophobic and sulfhydryl groups on the surface, which may contribute to the increased solubility of myosin [82]. Additionally, Chen et al. [83] found that L-His contains an imidazole ring that disrupts electrostatic interactions between myosin molecules, leading to alterations in the secondary and tertiary structures of the proteins, thereby facilitating myosin solubilization. This solubilizing effect enhances the mobility between protein molecules and provides a foundation for subsequent gel network formation.

Secondly, basic amino acids can form electrostatic interactions with negatively charged groups in proteins due to their positive charges [84]. Li et al. [85] found that L-Lys and L-Arg enhance the hydration capacity of myosin at low ionic strengths through cation-π interactions and electrostatic interactions with aromatic amino acid residues in myosin. These interactions promote the intermolecular cross-linking of proteins and stabilize gel networks [86]. The positively charged L-His can bind to negatively charged residues in myofibrillar proteins, disrupting the ionic linkages between proteins through electrostatic interactions. Additionally, L-His may extend the light-methylglobulin region, leading to the dissociation of myofibrillar filaments and an increase in solubility [87]. Furthermore, L-His can optimize the three-dimensional structure and gel properties of proteins by forming hydrogen bonds and hydrophobic interactions with myofibrillar proteins [88].

Finally, NaCl is a well-known pro-oxidant, as its increased ionic strength accelerates the rearrangement of the spatial structure of myofibrillar proteins. This exposure enhances their susceptibility to carbonylation [89]. In meat products, when basic amino acids act synergistically with salts, they can competitively bind to proteins, thereby inhibiting the pro-oxidant effects of NaCl. Furthermore, L-Lys and L-Arg exhibit a metal ion chelating activity, which further modulates the cross-linking patterns and antioxidant properties of proteins in meat products [90].

### 5.3. Moisture Distribution and Microstructure

The distribution and mobility of water in meat significantly contribute to essential qualities, such as juiciness, tenderness, firmness, and appearance [91]. The microstructure of the gel plays a crucial role in enhancing water retention in meat products; a dense and homogeneous microstructure results in improved WHC [28,92,93]. Lyutova and Reddy et al. [94,95] reported that arginine is effective in inhibiting protein aggregation and promoting protein unfolding. The unfolded structural state of myofibrillar proteins is vital for the formation of a continuous, homogeneous, and dense three-dimensional reticulated protein gel structure. This structure effectively traps and retains water within the protein gel matrix through capillary forces, thereby increasing the gel’s WHC. Zhang et al. [96] found that a combination of arginine and lysine effectively increases pH, reduces the gap between muscle fibers, and enhances the retention of fixed water in cooked marinated chicken breasts. Additionally, protein carbonylation can significantly impact the WHC of meat products [84], primarily by promoting the cross-linking of oxidized proteins. This process leads to the contraction of myofibrillar fibers and reduces the water retention by trapping water in a smaller space [85]. In contrast, the guanidino group in arginine and the imidazole group in lysine and histidine inhibit excessive oxidative cross-linking of myofibrillar proteins during thermal processing. This inhibition helps to reduce water loss and preserve the ordered structure of proteins [97]. In conclusion, basic amino acids cross-link with macromolecules, such as actin and myosin, in meat proteins, forming a more compact structural network that reduces protein oxidation, minimizes water loss, and maintains the porosity and elasticity of the meat.

## 6. Challenges and Development Trends of Basic Amino Acid-Based Salt Reduction Technology in Industrial Applications

Basic amino acids have garnered significant attention as a potential strategy for salt reduction technology; however, the widespread implementation of this technology in industrial settings still faces numerous challenges. The role of basic amino acids in flavor modulation is complex. While they can partially replace sodium salt and diminish the salty taste of food, excessive use may lead to an imbalance in overall flavor, which can affect consumer acceptance and potentially have adverse effects on human health [72]. For instance, high levels of histidine intake can result in anorexia and headaches, while excessive consumption of lysine may cause gastrointestinal discomfort [98]. Therefore, precisely controlling the amount of basic amino acids used to mitigate the negative impacts on food flavor has become a critical challenge for the successful industrial application of salt reduction technology.

Additionally, the cost of basic amino acids poses a significant barrier to their widespread use. Compared to conventional salts, the production costs of basic amino acids are high, particularly in large-scale manufacturing. Reducing these production costs and improving the economic feasibility of this technology remain key obstacles to its broader adoption [99]. Amino acids can be produced through various processes, including extraction from protein hydrolysates, chemical synthesis, and enzymatic or fermentation pathways utilizing microorganisms. In particular, fermentation engineering is emerging as one of the most promising methods for the commercial production of basic amino acids, owing to the application of advanced genetic engineering tools designed to maximize the yield, specificity, and productivity of target compounds [100]. During fermentation, the continuous monitoring of key parameters and process variables—such as inoculum quality, pH, feed rate, aeration intensity, and process temperature—is essential [101]. Efficient downstream processing and purification are critical for reducing the costs associated with amino acid production [102]. Amino acids are separated from the fermentation broth through centrifugation or filtration and subsequently purified using a chromatographic technique selected based on product characteristics, such as solubility, isoelectric point, and affinity for adsorbents. Researchers are exploring more efficient production methods, such as the fermentation of lysine and histidine using the aerobic, non-pathogenic Gram-positive bacterium *C. glutamicum* [102,103], as well as enhancing the yield and purity of basic amino acids through genetic engineering approaches [100].

In addition, the stability and shelf life of basic amino acids in food are critical factors that should not be overlooked. Different types of amino acids may degrade or undergo reactions in high-temperature, acidic, or basic environments, which can impact their functional properties and food safety. For instance, lysine can participate in the Maillard reaction during prolonged high-temperature treatments (e.g., frying, roasting, or baking), resulting in a loss of its biological activity [104]. Therefore, developing methods to enhance the stability of amino acids while preserving their activity during various food processing stages is a key research direction for promoting the widespread application of basic amino acid-based salt reduction technology.

Despite these challenges, basic amino acid salt reduction technology still possesses significant potential for widespread application. With the ongoing advancements in biotechnology and food processing technologies, it is anticipated that existing challenges will be mitigated through the optimization of processing methods, cost reduction, and stability enhancement, thereby promoting its broader adoption within the food industry. Additionally, the rising health consciousness among consumers, coupled with increasingly stringent government regulations regarding food safety and health, provides compelling incentives for the promotion of this technology.

## 7. Conclusions

This review serves as a reference for the application of basic amino acids in the salt reduction technology of meat products. Basic amino acids, such as lysine, arginine, and histidine, not only enhance the perception of saltiness and freshness but also improve the texture and WHC of meat products to a certain extent. Studies have demonstrated that the incorporation of basic amino acids in various types of meat products (e.g., sausages, hams, cured meats) can effectively reduce the amount of sodium salt used while maintaining or even enhancing the sensory quality of the products. Compared to traditional salt reduction strategies, basic amino acids improve the flavor perception in low-salt formulations by influencing the activation mechanisms of taste receptors and playing a crucial structural regulatory role in the meat system. These amino acids have been shown to interact with proteins and water molecules, enhancing the tenderness and juiciness of meat products, thereby compensating for the loss of flavor and texture associated with reduced salt content. Therefore, compared to traditional methods of reducing salt or using potassium salts as substitutes, basic amino acids offer a more comprehensive solution for salt reduction. This approach addresses the health needs of consumers while preserving or even enhancing the flavor and texture of meat products. Although basic amino acids present numerous advantages for salt reduction, their industrial application still encounters certain challenges. In conclusion, the use of basic amino acids for salt reduction in meat products is promising; however, further optimization of formulations, process improvements, and cost reductions are essential for more effective industrial implementation. Additionally, research should concentrate on developing more effective and safer salt reduction strategies utilizing basic amino acids to achieve a better flavor balance, enhance meat quality, and ensure food safety in low-salt environments.

## Figures and Tables

**Table 1:** Effect of the partial NaCl substitution by basic amino acids on the organizational and sensory properties of meat products.

Product	Salt Mixtures (%)	Organisational Changes	Effect of Sensory Properties	Main Findings	Reference
Salted chicken breast	C: 0% NaCl; 1% NaCl; 3% NaCl; SS1: 1% NaCl + 0.06% L-Lys SS2: 1% NaCl + + 0.06% L-Arg; SS3: 1% NaCl + 0.06% L-His	SS1 and SS2 increased protein solubility.	The addition of basic amino acids enhanced the WHC and texture.	The addition of basic amino acids improved solubility, emulsification, and gelation ability.	[53]
Frankfurter sausage	C: 1.75% NaCl; SS1: 0.875% NaCl + 0.1% L-Lys SS2: 0.875% NaCl + 0.3% L-Lys; SS3:0.875% NaCl + 0.5% L-Lys; SS4: 0.875% NaCl + 0.7% L-Lys	The gel network structure becomes denser and more uniform.	The addition of lysine enhanced the perception of salinity in the sodium-reduced group.	SS2 reduced cooking losses, improved emulsion stability and textural properties, and maintained high product quality with a 50% sodium reduction.	[54]
Fenneropenaeus chinensis	C: 2% NaCl; SS1: 0.5% NaCl SS2: 0.5% NaCl + 0.25% L-Arg; SS3: 0.5% NaCl + 0.5% L-Arg; SS4: 0.5% NaCl + 0.75% L-Arg; SS5: 0.5% NaCl + 1% L-Arg	SS4 has the lowest gel porosity and forms a denser gel network.	The L* values decreased. Hardness, elasticity, and chewiness increased.	0.75% L-Arg is regarded as the optimal level for enhancing the gelation properties of low-salt surimi.	[55]
Ham	C: 2.5% NaCl; SS1: 1.25% NaCl + 0.2% L-Lys; SS2: 1.25% NaCl + 0.4% L-Lys; SS3: 1.25% NaCl + 0.6% L-Lys; SS4: 1.25% NaCl + 0.8% L-Lys	The addition of L-Lys creates a finer network structure.	The SS4 group was comparable to the C group in taste and overall acceptability.	The optimal concentration of L-Lys for enhancing the physicochemical and organoleptic properties of salt-reduced ham is 0.8%.	[56]
Low-salt mixed shrimp and fish mince gel	C: 2.25% NaCl; SS1: 1% NaCl SS2: 1% NaCl + 0.3% STPP; SS3: 1% NaCl + 0.3% L-Lys; SS4: 1% NaCl + 0.6% L-Lys SS5: 1% NaCl + 0.3% L-Arg; SS6: 1% NaCl + 0.6% L-Arg	The gel network structure transitioned from a loose configuration to a homogeneous and dense form.	The addition of L-Lys and L-Arg increased the hardness, elasticity, and gel strength, particularly in the 0.3% Lys group.	L-Lys and L-Arg improved the strength and texture of low-salt mixed shrimp and fish surimi gels, surpassing traditional phosphates (STPPs). The optimal concentration of L-Lys was found to be 0.3%.	[57]
Beef patty	C: 0% NaCl, 1% NaCl, 2% NaCl, 3% NaCl, 4% NaCl, 5% NaCl; SS1: 1% NaCl +0.1%/0.5%/1%L-Lys; SS2: 1% NaCl +0.1%/0.5%/1%L-Arg; SS3: 1% NaCl +0.1%/0.5%/1%L-His	The hardness of beef patties decreased, while their elasticity increased with the addition of basic amino acids.	The 1% L-Lys and L-Arg groups exhibited the best organoleptic quality, with improved textural homogeneity and palatability.	1% L-Lys significantly inhibits the production of heterocyclic amines and improves the product quality.	[58]
Reduced-salt white leg shrimp (*Litopenaeus vannamei*) surimi gel	C: 1% NaCl; SS1: 0.5% NaCl and L-Arg (0.5%, 1.5%, 2.5%); SS2: 1% NaCl and 0.75% microbial transglutaminase (MTGase); SS3: 0.5% NaCl, 0.75% MTGase and L-Arg (0.5%, 1.5%, 2.5%)	The surimi developed a denser network structure, resulting in a decrease in porosity from 3.60% to 0.62%.	Synergistic treatment with L-Arg and MTGase significantly enhanced the firmness and chewiness of surimi.	The combination of L-Arg and MTGase reduced the NaCl content and significantly enhanced the gel strength, WHC, and textural properties of surimi.	[59]
Gna-type sausage	C: 2.5% NaCl; SS1: 1% NaCl + 1.5% KCl; SS2: 1% NaCl + 1.5% KCl + 1% Arg; SS3: 1% NaCl + 1.5% KCl + 0.2% His; SS4: 1% NaCl + 1.5% KCl + 1% Arg + 0.2% His	The addition of L-Arg and L-His enhanced emulsion stability and decreased water release.	The addition of L-Arg and L-His significantly improved the flavor and masked the metallic and astringent taste of KCl.	L-Arg and L-His improved the emulsion stability and textural properties by enhancing protein solubility and altering pH.	[60]

## Data Availability

The original contributions presented in this study are included in the article. Further inquiries can be directed to the corresponding author.
